# Unmasking the Underlying Causes: A Cross-Sectional Analysis of Mortality From Intentional and Unintentional Injuries in the United Arab Emirates

**DOI:** 10.7759/cureus.46567

**Published:** 2023-10-06

**Authors:** Hany A Zaki, Ahmed H Hamdi, Mohamed Elgassim, Bilal Albaroudi, Khalid Y Fadul, Amro Abdelrahman, Kaleem Basharat, Nood Dhafi R Al-Marri, Basel Elmegabar, Wael Abdelrehem Elnabawy Elsayed

**Affiliations:** 1 Emergency Medicine, Hamad Medical Corporation, Doha, QAT; 2 Emergency, Hamad Medical Corporation, Doha, QAT; 3 Medicine, Hamad General Hospital, Doha, QAT; 4 Medicine, Hamad Medical Corporation, Doha, QAT

**Keywords:** cross sectional studies, violence, suicide, mortality, injury, fall

## Abstract

The United Arab Emirates (UAE) has experienced substantial development in infrastructure and transportation in the last few decades. Although available evidence suggests that the incidence of injuries has considerably increased in the previous few years, there is a scarcity of literature that comprehensively analyzed the mortality due to different types of injuries in the UAE. Therefore, the current study was designed to report mortality due to various intentional and unintentional injuries in the UAE.

Secondary data was obtained from the World Health Organization (WHO) mortality database. We targeted injuries-specific mortality datasets. We applied a filter (UAE) to retrieve mortality data from unintentional and intentional injuries in the UAE. The latest data in the WHO mortality database was for 2020-21.

A total of 10,357 death records from the UAE were present in the WHO mortality database. The percentage of injury-specific deaths out of total deaths was 8.69% (n=900). Injury-specific mortality rate per 100,000 population was 9.09. The percentage of injury-specific deaths was higher for males (87.3%, n=786) and the age group 25-34 years (n=323, 35.9%).

Of the 900 injury-specific deaths, 449 (49.9%) were due to unintentional injuries, 216 (24.0%) were due to unintentional injuries, while the remaining (26.1%, n=235) deaths occurred due to Ill-defined injuries. More than half (53.7%, n=241) of unintentional injuries were because of road traffic injuries (RTIs) followed by fall (14.7%, n=66), exposure to mechanical forces (6.5%, n=29), drowning (6.0%, n=27) fire (1.1%, n=5), poisonings (1.1%, n=5), natural disasters (n=1, 0.2%) and other unintentional injuries (16.7%, n=75). More than three-quarters (83.3%, n=180) of intentional injuries were because of self-inflicted injuries while the remaining (16.7%, n=36) intentional injuries-specific deaths occurred due to violence.

Many deaths in the UAE occur due to unintentional and intentional injuries. RTIs and falls are the leading causes of unintentional injury-specific deaths, while self-inflicted injuries and violence are the leading causes of intentional injury-specific deaths.

## Introduction

Intentional or unintentional injuries are associated with incredible human suffering [1]. Despite advancements in medical care, severe intentional and unintentional injuries often result in disability or even death [2]. Evidence suggests that there has been a steep increase in mortality rates due to injuries in the last two decades [3]. Though the exact mechanisms that explain this steep increase are unknown, an increase in road traffic injuries (RTIs), falls, and violence in recent years may be responsible for the rapid increase in mortality due to injuries [4]. Globally, about 8% to 14% of deaths occur due to different types of injuries [5,6]. It is estimated that 16,000 people (almost five million per year) die daily from injuries [7].

Mortality due to injuries is not evenly distributed across different countries. Moreover, the causes of injury-related deaths also vary from one region to another [8]. For example, the highest rates of deaths associated with RTIs occur in the Middle East region. In contrast, the highest violence-related deaths are reported from Asian and African regions [9-12]. This signifies the need for a comprehensive analysis of mortality rates and their causes in different world regions.

United Arab Emirates (UAE), a country in the Middle East, has experienced substantial development in infrastructure and transportation in the last few decades [13,14]. This rapid development has increased the incidence of intentional and unintentional injuries. For example, with the swift growth of the automobile industry and transportation, there is a significant increase in RTIs [15,16]. Likewise, due to the increase in civil construction, fall-related injuries have been increased [17]. Moreover, the rapid infrastructural, economic, and technological development have impacted the behaviors and mental health of residents of the UAE, due to which intentional injuries such as interpersonal violence and self-harm have dramatically increased [18,19]. Although available evidence suggests that the incidence of injuries has considerably increased in the last few years, a scarcity of literature comprehensively analyzes the mortality due to different types of injuries in the UAE. Therefore, the current study was designed to report mortality due to different intentional and unintentional injuries in the UAE.

## Materials and methods

Our research utilized secondary data from the World Health Organization's (WHO) renowned mortality database. The WHO mortality database stands as a comprehensive repository, meticulously collated over the years, that chronicles mortality rates across the globe. It provides a window into the myriad causes of death, capturing data from diseases, ailments, and other conditions that afflict populations worldwide.

While encompassing the vast database scope, our study zeroed in on a particular segment: injury-related mortality. This choice was driven by our key objective of delving deep into the nuanced causes behind deaths due to injuries. For ease of reference and navigation, the WHO has allocated a dedicated webpage for this section, aptly titled “Injuries,” and it can be accessed directly through the URL “Injuries (who.int).”

Taking the granularity a step further, the WHO, in its effort to provide a clearer picture of injury-related mortalities, has segmented the injury data into two distinct categories. The first, known as unintentional injuries, encapsulates those injuries that occur without deliberate intent. These could range from accidents, falls, or any other unexpected events leading to injury. This segment is detailed on their webpage “Unintentional injuries (who. int).” The second category is intentional injuries, which, as the name suggests, includes injuries stemming from deliberate acts such as assaults, self-harm, or other intentional actions. Details about this category can be found in “Intentional injuries (who.int).”

For regional specificity and to hone in on data most relevant to our study, we deployed a filter for the “United Arab Emirates.” This filtering process enabled us to distill the vast amount of global data to mortality statistics related to the UAE, ensuring our research remained geographically pertinent. By doing so, we were able to gain insights specifically into unintentional and intentional injury-related mortalities within the context of the UAE. Finally, Chi-square was applied to find the association between gender, age group, and cause of injury, where applicable. P-value<0.05 was considered significant.

## Results

**Figure 1:**
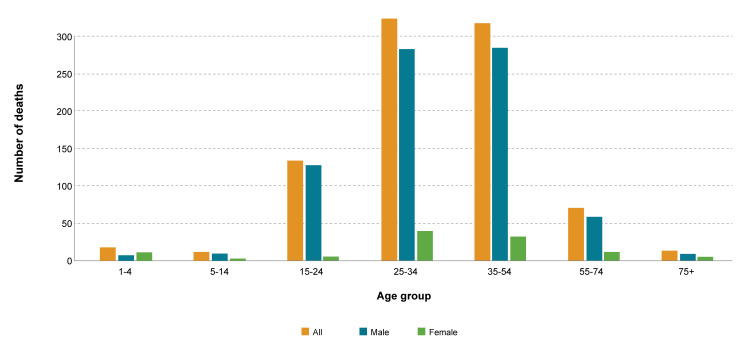
Mortality due to injury-specific causes in men and women of different age groups The orange color overall injury specific deaths, blue show mortality in men, and green show mortality in women. From the visual presentation, it is clear that mortality was highest among men of age groups 25-34 years and 35-54 years.  Similarly, women of age groups 25-34 years and 35-54 years also have higher mortality compared to other age groups. The third age group with high mortality was 15-24 years followed by 55-74 years groups. The figure explicitly conveys the message that injury-specific deaths were more common in young men. Moreover, it is clear that mortality was lowest in age groups 1-4 years, 5-14 years, and 75+ years. Similarly, in all age groups female have less mortality compared to their counterpart male.

A total of 10,357 death records from the UAE were present in the WHO mortality database. The percentage of injury-specific deaths out of total deaths was 8.69% (n=900). Injury-specific mortality rate per 100,000 population was 9.09. The percentage of injury-specific deaths was higher for males (87.3%, n=786) than females (12.7%, n=114). Injury-specific deaths were highest (n=323, 35.9%) for age group 25-34 years, followed by age group 35-54 years (n=317, 35.2%), 15-24 years (n=134, 14.9%), ≥ 55 years (n=85, 9.5%), and ≤ 14 years (n=41, 4.5%). Figure 1 shows the mortality due to injury-specific causes. There was no significant association (P>0.05) between age group and gender in people with injury-specific deaths.

Unintentional injuries

**Figure 2:**
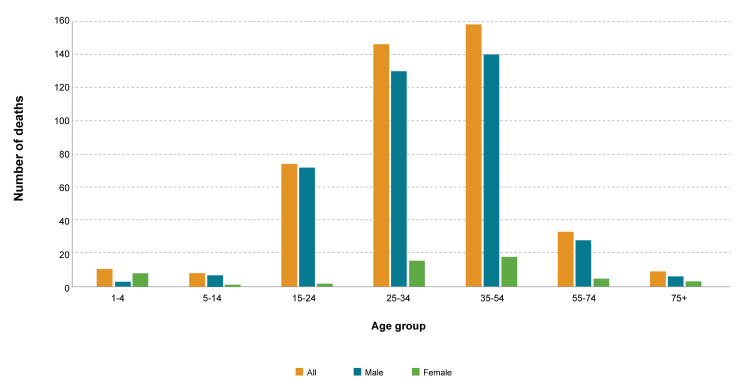
Mortality due to unintentional injury-specific causes in men and women of different age groups The orange color overall injury-specific deaths, blue show mortality in men, and green show mortality in women. From the visual presentation, it is clear that mortality was highest among men of age groups 25-34 years and 35-54 years.  Similarly, women of age groups 25-34 years and 35-54 years also have higher mortality compared to other age groups. The third age group with high mortality was 15-24 years followed by 55-74 years groups. The figure explicitly conveys the message that unintentional injury-specific deaths were more common in young men. Moreover, it is clear that mortality was lowest in age groups 1-4 years, 5-14 years, and 75+ years. Similarly, in all age groups female have less mortality compared to their counterpart male.

Of the 900 injury-specific deaths, 449 (49.9%) were due to unintentional injuries. The percentage of unintentional injuries-specific deaths out of total deaths was 4.33%. Unintentional injuries-specific mortality rate per 100,000 population was 4.53. The percentage of unintentional injury-specific deaths was higher for males (87.3%, n=392) than females (12.7%, n=57). Unintentional injuries-specific deaths were highest (n=158, 35.2%) for the age group 35-54 years, followed by the age group 25-34 years (n=146, 32.5%), 15-24 years (n=74, 16.5%), ≥ 55 years (n=42, 9.3%) and ≤ 14 years (n=29, 6.5%). Figure 2 showed a diagram illustrating the Mortality due to unintentional injuries, where there was no significant association (P>0.05) between age group and gender in people with unintentional injury-specific deaths.

Of the 449 deaths due to unintentional injuries, more than half (53.7%, n=241) were because of RTIs. The percentage of RTI-specific deaths out of total deaths was 2.32%. RT-specific mortality rate per 100,000 population was 2.43. Other causes of unintentional injuries-specific deaths were falls (14.7%, n=66), exposure to mechanical forces (6.5%, n=29), drowning (6.0%, n=27), fire (1.1%, n=5), poisonings (1.1%, n=5), natural disasters (n=1, 0.2%) and other unintentional injuries (16.7%, n=75). The causes of mortality due to unintentional injuries are described in Table 1.

**Table 1:** Causes of mortality due to unintentional injuries Mortality due to different types of unintentional injuries depicted in the rows, including road traffic accident, fall, exposure to mechanical forces, drowning, fire, poisonings, natural disasters, and other unintentional injuries. In the columns, the percentage of deaths out of total reported deaths, mortality rate, percentage due to different types of unintentional injuries, and gender-wise distribution are shown. RTI; Road Traffic Injuries, n; Number, %; Percentage.

Cause of injury	Percentage of cause-specific deaths out of total deaths (n=10357)	Mortality rate per 100 000 population	Percentage (frequency) of cause-specific deaths out of unintentional injuries-related deaths (n=449)	Percentage (frequency) of cause-specific deaths in male (n=392)	Percentage (frequency) of cause-specific deaths in female (n=57)
RTI	2.32%	2.43	53.7% (241)	89.2% (215)	10.8% (26)
Fall	0.63%	0.67	14.7% (66)	81.8% (54)	18.2% (12)
Exposure to mechanical forces	0.28%	0.29	6.5% (29)	93.1% (27)	6.9% (2)
Drowning	0.26%	0.27	6.0% (27)	88.9% (24)	11.1% (3)
Fire	0.04%	0.05	1.1% (5)	80.0% (4)	20.0% (1)
Poisonings	0.04%	0.05	1.1% (5)	80.0% (4)	20.0% (1)
Natural disasters	0.009%	0.01	0.2% (1)	100% (1)	0 (0)
Other unintentional injuries	0.72%	0.75	16.7% (75)	84.0% (63)	16.0% (12)

Intentional injuries

**Figure 3:**
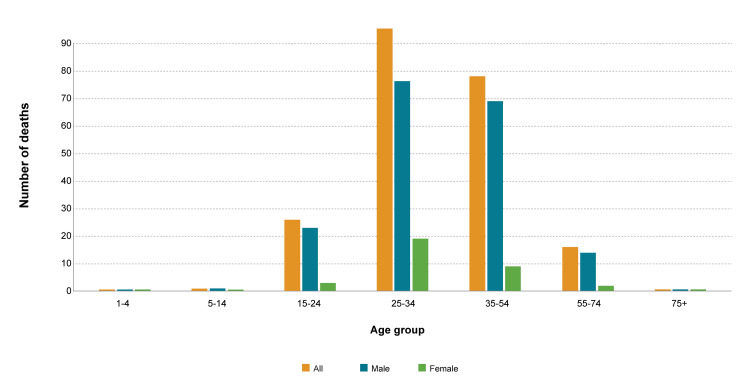
Mortality due to intentional injury-specific causes in men and women of different age groups The orange color overall injury-specific deaths, blue show mortality in men, and green show mortality in women. From the visual presentation, it is clear that mortality was highest among men of age groups 25-34 years and 35-54 years.  Similarly, women of age groups 25-34 years and 35-54 years also have higher mortality compared to other age groups. The third age group with high mortality was 15-24 years followed by 55-74 years groups. The figure explicitly conveys the message that intentional injury-specific deaths were more common in young men. Moreover, it is clear that mortality was lowest in age groups 1-4 years, 5-14 years, and 75+ years. Similarly, in all age groups female have less mortality compared to their counterpart male.

Of the 900 injury-specific deaths, 216 (24.0%) were due to intentional injuries. The percentage of unintentional injuries-specific deaths out of total deaths was 2.08%. Unintentional injuries-specific mortality rate per 100,000 population was 2.18. The percentage of intentional injury-specific deaths was higher for males (84.7%, n=183) than females (15.3%, n=33). Intentional injuries-specific deaths were highest (n=95, 44.0%) for age group 25-34 years, followed by age group 35-54 years (n=78, 36.1%), 15-24 years (n=26, 12.0%), ≥ 55 years (n=16, 7.4%) and ≤ 14 years (n=1, 0.5%). Figure 3 showed a diagram illustrating the mortality due to intentional injuries, where there was no significant association (P>0.05) between age group and gender in people with intentional injury-specific deaths.

More than three-quarters of the 216 deaths due to intentional injuries (83.3%, n=180) were because of self-inflicted injuries. The percentage of deaths due to self-inflicted injuries out of total deaths was 1.73%. The mortality rate per 100,000 population due to self-inflicted injuries was 1.81. Another cause of intentional injury-specific deaths was violence (16.7%, n=36). The causes of mortality due to intentional injuries are described in Table 2.

**Table 2:** Causes of mortality due to intentional injuries Mortality is due to different types of intentional injuries depicted in the rows, including self-inflicted injuries and violence. In the columns, the percentage of deaths out of total reported deaths, mortality rate, percentage due to different types of unintentional injuries, and gender-wise distribution are shown. n; Number, %; Percentage.

Cause of injury	Percentage of cause-specific deaths out of total deaths (n=10357)	Mortality rate per 100 000 population	Percentage (frequency) of cause-specific deaths out of intentional injuries-related deaths (n=216)	Percentage (frequency) of cause-specific deaths in male (n=183)	Percentage (frequency) of cause-specific deaths in female (n=33)
Self-inflicted injuries	1.73%	1.81	83.3% (180)	86.1% (155)	13.9% (25)
Violence	0.34%	0.36	16.7% (36)	77.8% (28)	22.2% (8)

Ill-defined injuries

**Figure 4:**
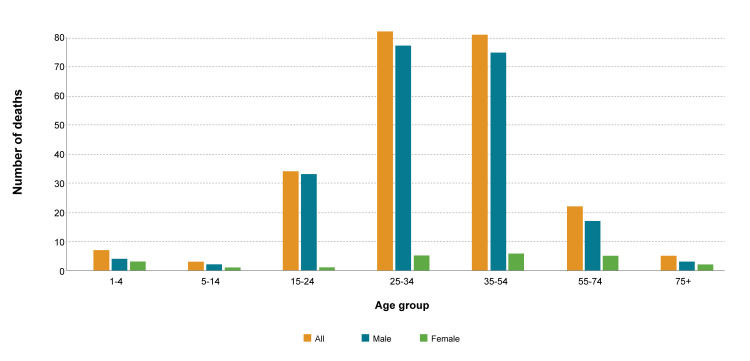
Mortality due to ill-defined injuries-specific causes in men and women of different age groups The orange color overall injury-specific deaths, blue show mortality in men, and green show mortality in women. From the visual presentation, it is clear that mortality was highest among men of age groups 25-34 years and 35-54 years.  Similarly, women of age groups 25-34 years and 35-54 years also have higher mortality compared to other age groups. The third age group with high mortality was 15-24 years followed by 55-74 years groups. The figure explicitly conveys the message that ill-defined injury-specific deaths were more common in young men. Moreover, it is clear that mortality was lowest in age groups 1-4 years, 5-14 years, and 75+ years. Similarly, in all age groups female have less mortality compared to their counterpart male.

Of the 900 injury-specific deaths, almost one quarter (26.1%, n=235) have occurred due to Ill-defined injuries. The percentage of ill-defined injuries-specific deaths out of total deaths was 2.27%. The ill-defined injuries-specific mortality rate per 100,000 population was 2.38. The percentage of intentional injury-specific deaths was higher for males (89.8%, n=211) than females (10.2%, n=24). Ill-defined injuries-specific deaths were highest (n=82, 34.9%) for the age group 25-34 years, followed by the age group 35-54 years (n=81, 34.5%), 15-24 years (n=34, 14.5%), ≥ 55 years (n=27, 11.5%), and ≤ 14 years (n=11, 4.6%). Figure 4 showed there was no significant association (P>0.05) between age group and gender in people with ill-defined injuries-specific deaths.

## Discussion

Moderate to severe injuries can lead to deaths, and evidence suggests that causes of injury-specific deaths vary across different regions [20,21]. The current study reported mortality rates due to various intentional and unintentional injuries in the UAE. The findings suggest that almost 8.6% of deaths occurred due to injury-specific causes. This is following global statistics related to injury-specific deaths. According to WHO, 8% of deaths globally occur due to injuries [22], though the statistics vary from country to country. Though the remaining 92% of deaths occur due to other causes, there is a consensus that mortality associated with injury-specific causes has much more debilitating effects on society than other causes of mortality [1]. The sufferings due to injury are not limited only to the person but also impact the family, friends, and community. Most frequently, these injury-specific deaths are sudden, thus leading to devastating consequences [23].

The current study's findings reported that the percentage of injury-specific deaths was much higher for males (87.3%) than females (12.7%). UAE is a male-dominated society, and males are more exposed to injury-specific causes than females. Typically, males take on physically intensive roles like construction, making them more susceptible to injuries [24]. Similarly, since men predominantly undertake driving roles, they are at a higher risk of RTIs. A literature search also revealed that injury-specific deaths are more common in males than females [25]. Results of the current study suggest that injury-specific deaths were highest in the age group 25-34 years and 35-54 years. Again, there is consensus in the literature that injuries are highly prevalent in young age [26]. Individuals are active at a young age, and thus, chances of injury are higher at a young age. Moreover, evidence suggests that young people in the productive years of their lives are more active because of the demands of their professional growth. Furthermore, young individuals are eager to take physical risks, increasing the chances of injury [27].

The current study showed that about half of the injury-specific deaths occurred due to unintentional injuries. Globally, unintentional injuries are one of the leading causes of mortality [28]. Of the unintentional injuries, RTIs are the comments cause of mortality. There are consistent reports in the literature that RTIs have dramatically increased in the UAE due to increased vehicle numbers, aggressive driving behaviors, and non-compliance with road safety protocols [29]. Previous studies have reported that fatality rates associated with RTIs in Middle Eastern countries are much higher than in most developed countries. Though there is a scarcity of high-quality evidence that explains the exact factors that might be responsible for high mortality associated with RTIs in UAE, from existing literature, it can be deduced that overspeeding, delay in the provision of proper emergency services, lack of awareness about road safety protocols and aggressive behaviors are some of the factors that may be responsible for high mortality due to RTIs in UAE [30,31].

The second leading cause of unintentional injury-specific deaths was falls. Though we could not explore the underlying causes of falls, previous research studies reported that fall-related injuries in the UAE are more common in construction workers [17]. Literature suggests that construction workers often do not follow occupational safety measures and thus are at risk of fall-related injuries. Sports, recreational activities, and household chores are some of the underlying causes of on-ground-related falls. Moreover, fall from the tree, polls, buildings, and bridges are less commonly reported cause of fall-related injuries [32,33]. Exposure to mechanical forces is another leading cause of mortality in the UAE. Literature suggests that industrial and construction workers are mostly exposed to mechanical forces [16].

The current study's findings revealed that intentional injuries were less common than unintentional injuries. However, almost one-quarter of injury-specific deaths in the UAE occur due to intentional injuries. The two leading causes of intentional injuries-specific deaths were self-inflicted injuries and violence. There are consistent reports in the literature that behavioral and mental health issues are responsible for most intentional injuries [34,35]. Previous studies conducted in the UAE also reported that behavioral and mental health has increased in recent years, increasing the risk of intentional injuries, including self-harm and violence [15,36]. Though the exact mechanisms that explain the drastic increase in behavioral and mental health issues in UAE and the subsequent increase in intentional injuries are not precise, nonetheless, available evidence suggests that domestic issues, financial matters, professional challenges, cultural taboos, and social restrictions are some of the underlying factors that might be responsible for most of the intentional injuries [37,38].

The current study is one of the first to comprehensively analyze and report mortality from different intentional and unintentional injuries in the UAE. Nonetheless, the recent study has some limitations. To begin with, the current study utilized secondary data, and we could not trace the methods through which data was collected. Moreover, the current study reported findings for the whole UAE, and it was impossible to compare injury-specific mortality in different states and regions. Finally, the generalizability of the present study's findings to other countries may be questionable. Considering these limitations, we recommend high-quality multinational population-based surveys to determine the mortality burden due to intentional and unintentional injuries.

## Conclusions

A large proportion of deaths in the UAE occur from injury-specific causes. Mortality due to injury-specific causes is highly prevalent in males compared to females. Young individuals, particularly those in their third or fourth decade, are more prone to injury-specific deaths. About half of the injury-specific deaths occurred due to unintentional injuries. RTIs were the leading cause of unintentional injuries-specific deaths, followed by falls, exposure to mechanical forces, drowning, fire, poisonings, natural disasters, and other unintentional injuries. Though intentional injury-specific deaths were less common than unintentional injuries, almost a quarter of the injury-specific deaths were due to intentional injuries. Self-inflicted injuries and violence were the leading causes of intentional injury-specific deaths.

Our study underscores the pressing need for comprehensive research on injury-induced mortality. We strongly advocate for the execution of high-quality, multinational, population-based studies to accurately determine the mortality burden of intentional and unintentional injuries. Such studies would provide a more profound grasp of the global ramifications of these injuries and set the stage for informed interventions and policy decisions. By encompassing multiple countries, we can ensure a broad representation, accounting for the myriad cultural, socioeconomic, and environmental differences, offering a holistic perspective.
